# Randomized controlled trial of a health plan-level mood disorders psychosocial intervention for solo or small practices

**DOI:** 10.1186/s40359-014-0048-x

**Published:** 2014-11-13

**Authors:** Amy M Kilbourne, Kristina M Nord, Julia Kyle, Celeste Van Poppelen, David E Goodrich, Hyungjin Myra Kim, Daniel Eisenberg, Hyong Un, Mark S Bauer

**Affiliations:** VA Center for Clinical Management Research (CCMR), VA Ann Arbor Healthcare System, 2215 Fuller Road, Mailstop 152, Ann Arbor, MI 48105 USA; Department of Psychiatry, University of Michigan Medical School, North Campus Research Complex, 2800 Plymouth Road, Building 16, Ann Arbor, MI 48109-2800 USA; Department of Health Management and Policy, School of Public Health, University of Michigan, 1415 Washington Heights, Ann Arbor, MI 48109-2029 USA; Aetna Healthcare, 980 Jolly Road, Blue Bell, PA 19422 USA; Center for Healthcare Organization and Implementation Research, VA Boston Healthcare System 152M, 150 South Huntington Avenue, Boston, MA 02130 USA; Department of Psychiatry, Harvard Medical School, 2 West, Room 305, 401 Park Drive, Boston, MA 02215 USA

**Keywords:** Depression, Health behavior change, Care management, Health plans

## Abstract

**Background:**

Mood disorders represent the most expensive mental disorders for employer-based commercial health plans. Collaborative care models are effective in treating chronic physical and mental illnesses at little to no net healthcare cost, but to date have primarily been implemented by larger healthcare organizations in facility-based models. The majority of practices providing commercially insured care are far too small to implement such models. Health plan-level collaborative care treatment can address this unmet need. The goal of this study is to implement at the national commercial health plan level a collaborative care model to improve outcomes for persons with mood disorders.

**Methods/Design:**

A randomized controlled trial of a collaborative care model versus usual care will be conducted among beneficiaries of a large national health plan from across the country seen by primary care or behavioral health practices. At discharge 344 patients identified by health plan claims as hospitalized for unipolar depression or bipolar disorder will be randomized to receive collaborative care (patient phone-based self-management support, care management, and guideline dissemination to practices delivered by a plan-level care manager) or usual care from their provider. Primary outcomes are changes in mood symptoms and mental health-related quality of life at 12 months. Secondary outcomes include rehospitalization, receipt of guideline-concordant care, and work productivity.

**Discussion:**

This study will determine whether a collaborative care model for mood disorders delivered at the national health plan level improves outcomes compared to usual care, and will inform a business case for collaborative care models for these settings that can reach patients wherever they receive treatment.

**Trial registration:**

ClinicalTrials.gov Identifier: NCT02041962; registered January 3, 2014.

## Background

A recent report from the Department of Health and Human Services highlighted the prevalence, morbidity, and cost associated with clusters of co-occurring chronic conditions, both physical and mental (U.S. Department of Health and Human Services 2011). Evidence suggests that collaborative care models (**CCMs**) are effective in treating chronic medical and mental illnesses at little to no net healthcare cost (A National Agenda for Research in Collaborative Care. June 2011; Woltmann et al. 2012; Bodenheimer et al. 2002; Wagner et al. 1996; Coleman et al. 2009a). CCMs typically consist of patient self-management skill enhancement, expert decision support to providers via evidence-based practice guidelines, and enhanced access and continuity via care managers (Bauer 2001; Bauer et al. 2001). CCMs will become increasingly important as healthcare delivery systems evolve into accountable care organizations (Fisher et al. 2009; Shortell & Casalino 2010), thereby taking on broader responsibility for care coordination and quality while bearing financial risk for complex, chronic conditions. CCMs can provide either the foundation of, or an annex to, emerging medical home methodologies as well (Nutting et al. 2009; Carrier et al. 2009; Rittenhouse et al. 2008).

To date, evidence-based CCMs have primarily been implemented at the facility level in publically funded settings (A National Agenda for Research in Collaborative Care. June 2011; Woltmann et al. 2012; Coleman et al. 2009b; Collins et al. 2010) or within integrated healthcare systems (Rittenhouse et al. 2010; Casalino et al. 2003; Druss et al. 2010; Katon et al. 2010) but not in smaller practices (Bauer et al. 2012a). However, the majority (between 50-70%) of patients receive care from network-model health plans and within small practices of less than 20 providers (Bauer et al. 2012a; Findlay 1999). These smaller practices are less likely to be able to implement care management processes (Rittenhouse et al. 2011). The few trials involving CCMs delivered remotely (off-site) recommend a combination of rapport-building telephone care manager-patient contacts and personally tailored self-management resources (Datto et al. 2003; Hunkeler et al. 2000; Ludman et al. 2007a; Lynch et al. 2004; Mohr et al. 2005; Oslin et al. 2003; Ransom et al. 2008; Simon et al. 2000; Simon et al. 2009; Stein et al. 2007; Tutty et al. 2005; Lynch et al. 1997; Pariser & O'Hanlon 2005).

The goal of this study is to implement at the national level through a commercial health plan a CCM that is designed to improve outcomes for persons with mood disorders. Mood disorders represent optimal tracer conditions with which to improve management strategies using CCMs for individuals with multiple chronic conditions. Chronic mood disorders (e.g., bipolar disorder, depression) are common and are associated with extensive functional impairment, medical comorbidity, and personal and societal costs (Bauer 2008). National studies of the U.S. population estimate the lifetime prevalence for bipolar spectrum disorders as 6.4% (Judd & Akiskal 2003; Merikangas et al. 2007) and 16.6% for major depressive disorder (Kessler & Wang 2008). Quality of care is suboptimal for both chronic medical (Institute of Medicine 2003; Lopez & Murray 1998) and mental (Boardman 2006; Busch et al. 2004; Druss et al. 2000; Druss et al. 2002; Hogan 2003; Leslie & Rosenheck 2004; Leslie & Rosenheck 2003; Sernyak et al. 2003) disorders, underscoring the need for coordinated, comprehensive care. While unipolar depression is more common, patients with bipolar disorder incur the most health care costs of any mental illness (Peele et al. 2003). Up to 70% of direct treatment costs for mood disorders are generated outside the mental health sector, notably in primary care (Bryant-Comstock et al. 2002; Simon & Unutzer 1999; Dilsaver 2011). In response to extremely high costs and high disease burden associated with mood disorders, CCMs have been found to be effective in reducing symptom burden and improving health-related quality of life for depression (Woltmann et al. 2012; Gilbody et al. 2006; Badamgarav et al. 2003; Unutzer et al. 2008) and bipolar disorder (Bauer 2001; Bauer et al. 2001; Bauer et al. 2006a; Bauer et al. 2006b; Kilbourne et al. 2008; Simon et al. 2006) in separate studies and are now recommended in practice guidelines (American Psychiaric Association 2002; Yatham et al. 2009).

### Aims and objectives

The primary aim of this study is to determine whether individuals with mood disorders from practices treated with a health plan-level CCM demonstrate improved health outcomes in 12 months compared to those who receive usual care.

Our primary hypotheses are that compared to usual care, the CCM will result in 1) decreased mood symptoms in 12 months based on the nine-item Patient Health Questionnaire (PHQ-9), or 2) improved mental health-related quality of life based on the Short Form Health Survey (SF-12). Our secondary hypotheses are that patients receiving CCM versus usual care will have 1) reduced probability of hospitalization, 2) improved guideline-concordant care (e.g., mood disorders treatment, cardiometabolic monitoring), and 3) improved work productivity within 12 months.

Exploratory aims of this study are to support subsequent CCM dissemination by identifying key patient characteristics associated with CCM engagement and outcomes, to estimate the costs of CCM versus usual care, and assess the incremental costs per difference in patient-level utility associated with CCM versus usual care over a 24-month period.

## Methods

This single-blind randomized controlled effectiveness trial will compare patients receiving the **CCM** for mood disorders versus usual care. The population of interest will be Aetna adult enrollees and family members (beneficiaries) across the country hospitalized for an episode of unipolar depression or bipolar disorder. The University of Michigan Medical School Institutional Review Board approved this study with a waiver of documentation of written consent (IRBMED HUM00073753) and the study was registered with ClinicalTrials.gov on January 3, 2014 (NCT02041962). All participants will provide verbal informed consent to the Aetna care manager, and will receive a mailed copy of the consent for their records.

### Setting

The CCM will be implemented by providers employed at Aetna Behavioral Health for beneficiaries from across the country who are hospitalized for depression or bipolar disorder. Aetna health plan is the fifth largest healthcare insurer in the country, providing benefits through employers in all 50 states. Serving approximately 12 million covered lives, with 244,971 providers filing claims within the past year, Aetna Behavioral Health has made the development and implementation of CCMs a top priority. Among its enrollees, over 90% were seen in solo or small practices of less than 4 providers (Bauer et al. 2012b).

### Participant selection

The Aetna care manager will recruit participants by first screening and consenting them based on near-real time information of recent hospitalizations. At hospitalization, Aetna is notified for (pre)authorization, typically before or within 48 hours of admission. The care manager will be notified about patient hospitalizations via the Aetna care management registry and will contact the potential participant by phone, screen for eligibility, and obtain informed consent and authorization to release information to the research team and to coordinate care with their providers. Because patients have not been randomized at this point, Aetna care managers will be blind to treatment assignment at baseline.

Patient inclusion criteria as determined by the care managers include:

- Adult patients age 21 years or older from the contiguous United States (lower 48 states)
- Currently covered by Aetna’s HMO or preferred provider products (for whom Aetna provides mental and medical inpatient, outpatient, and pharmacy benefits) for at least 6 months
- Recent (past 6-month) hospitalization for an acute psychiatric or partial hospital unit with a manic or depressive episode and confirmation of mood disorder diagnosis in the medical record (presence of one inpatient or two outpatient designated by International Classification of Diseases, Ninth Revision, Clinical Modification (ICD-9-CM) diagnostic codes: 296.1×—296.8× in previous 6 months) (Kilbourne et al. 2008).
- Ability to speak and read English and provide informed consent.

### Study design and randomization

**Figure 1:**
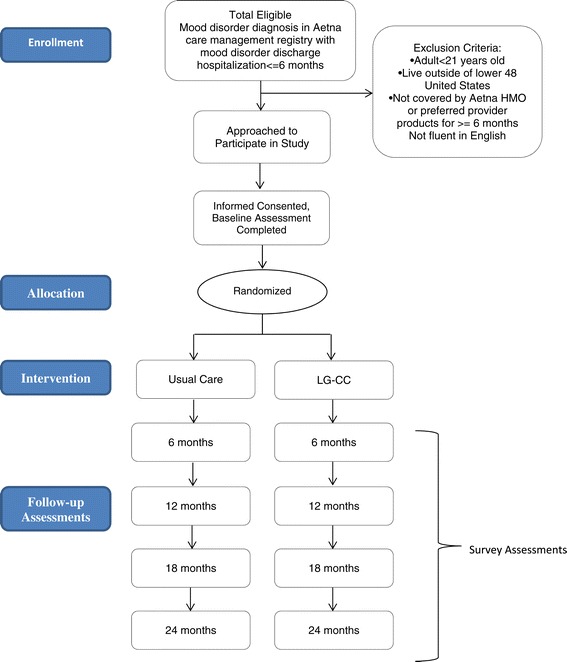
**Consort flow diagram.**

As displayed by the Consort diagram in Figure 1, patients will be randomized to CCM or usual care using a computer generated algorithm that will stratify randomization by diagnosis at hospitalization discharge (unipolar disorder, bipolar disorder). The Care Manager, prior to randomization, will ascertain baseline information from enrolled and eligible patients via a brief survey (see Outcomes section below for questions). Remaining outcomes assessments will be completed by a separate research assistant who is not employed by the health plan, and will also be blinded to randomization assignment. The Care Manager will be trained to conduct baseline assessments by study staff on initiating calls across time zones and in human subjects risk reduction procedures used in prior studies (Bauer et al. 2001; Bauer et al. 2006a) that will minimize risk while not compromising study internal validity (Bauer et al. 2001).

### Intervention

Patients randomized to the usual care arm will receive standard care from their practice provider, but none of the CCM components from the plan-level care manager.

Patients randomized to the intervention will receive the plan-level CCM in addition to their usual care from their provider. The CCM intervention will be delivered over a twelve-month period, and include an initial contact with patient enrolled in the CCM arm, 10 weekly self-management sessions, ongoing care management, and dissemination of guidelines and follow-up with patients’ principal healthcare providers regarding clinical issues.

The CCM is based on the Life Goals Collaborative Care program (Table 1), which was found in several randomized controlled trials to improve physical and mental health outcomes for persons with mood disorders (3). Life Goals Collaborative Care components delivered by the Care Manager include the Life Goals self-management program, care management via ongoing contacts to facilitate flow of current clinical status information between patients and their principal healthcare providers, and dissemination of evidence-based clinical practice guidelines for mood disorders to the providers.

**Table 1:** **Mood disorders CCM core elements**

**Life goals self-** **management program**	**10 weekly 30**-**min telephonic sessions** utilizing the Life Goals program that include core modules covering management of depressive symptoms and additional modules utilized as clinically indicated (e.g., mania, wellness, foods and moods, physical activity, substance use, anxiety, psychosis, anger/irritability):
	Week 1: Introduction – Understanding your mental health and stigma
	Week 2: Introduction (Continued) – Personal values and Life Goals
	Week 3: Identifying personal symptoms of depression
	Week 4: Identifying triggers and responses to depression
	Week 5: Development of personal action plan for coping with depression
	Week 6: Optional Session 1
	Week 7: Optional Session 2
	Week 8: Optional Session 3
	Week 9: Managing Your Care – Provider visit preparation
	Week 10: Plan for continuing to work toward your Life Goals
**Access/** **continuity/** **care management**	**12 monthly patient telephone contacts for 1 year** (in addition to the self-management program) to trouble-shoot self-management issues and summarize clinical status
	• Ad hoc contacts at either care manager or participant initiation based on clinical or other concerns, including response to participants within one business day
	• “In-reach” to treating clinicians for hospitalization, ER visits, or specialty consultation
	• Collaboration with family as permitted
	• Resource referral as needed
**Provider decision support**	**Provider contacts**
	• Same content as clinic-based CCM
	• Guidelines disseminated where appropriate based on AHRQ depression in primary care and APA bipolar guidelines

The Life Goals self-management program includes psychoeducation based on Social Cognitive Theory, emphasizing brief Motivational Interviewing and cognitive-behavioral techniques, particularly behavioral activation, to address symptom management and problem-solving skills. Ten core modules (see Table 1) will be delivered over 10 weekly telephonic sessions of 30 minutes (Ludman et al. 2007a; Ludman et al. 2007b). The care manager will deliver the 10 Life Goals self-management program modules via telephone and using a workbook mailed to patients that contains modules, exercises, and other information on mood disorders (Kilbourne et al. 2008; Bauer et al. 2008). For patients with bipolar disorder, at least one of the modules will focus on coping with manic symptoms, while patients with unipolar depression will also receive an additional module on depressive symptoms (Ludman et al. 2007b).

The care management calls with patients will continue on a monthly basis for up to a year after the self-management phone sessions are completed, with as-needed phone contacts made to follow up in the event of a hospitalization or emergency room visit. Imminent risk (suicidal or assaultive ideation, significant medication toxicity) will be managed via protocols used in prior studies (Bauer et al. 2001; Bauer et al. 2006a; Kilbourne et al. 2008). The care manager will also contact the patients’ providers on an as needed basis such as in the event of hospitalization, emergency room visit, or development of a new clinical issue, as well as to cue them if there is a crisis encounter (e.g., suicidality). It is important to note that, as in our prior studies (Bauer et al. 2006a; Bauer et al. 2006b; Kilbourne et al. 2008; Simon et al. 2006; Bauer et al. 1997), the CCM is designed to supplement care and does not replace or control provider decision-making. Clinical decision-making remains in the hands of the provider. The CCM therefore enhances care processes by: (a) enhancing patient skills to facilitate treatment participation, (b) offering the provider timely information, and (c) outlining situation-specific evidence-based treatment options.

Fidelity to the CCM will be maximized via a 2-day training session for the care manager, as well as regular ongoing calls with the care manager and training to review enrollment progress and review session content delivered to patients randomized to the CCM.

### Measures and outcomes

Baseline and outcomes data will be ascertained from patients (including surveys, medical record reviews, and claims data) (Table 2). A 30-minute quantitative survey will be used to ascertain information from patients at baseline, 6, 12, 18, and 24 months thereafter. Patient data will be entered into a web-based data management system. The baseline assessment will include a brief survey on demographics and baseline outcomes measures for mood symptoms, quality of life, and employment and work productivity. Follow-up surveys will include questions on mood symptoms, quality of life, and employment and work productivity. There is the possibility that enrolled patients may inadvertently disclose their randomization status with the RA, and this will be mitigated through RA training (e.g., minimizing leading questions that would disclose treatment assignment).

**Table 2:** **Primary and secondary outcomes and measures**

**Aims**	**Key measures**	**Source**
Aim 1. primary outcomes	Mood symptoms: PHQ-9	Patient survey
	Mental health-related quality of life-SF-12 (MCS)	Patient survey
Aim 1. secondary outcomes	Hospitalizations	Aetna claims data
	Guideline concordant care:	Medical record/claims
	Mood disorders: % receiving guideline-concordant antidepressants (if unipolar depression) or guideline-concordant anti-manic treatment (bipolar disorder dx) in 6-month period	
	Cardiometabolic monitoring: % receiving lipid profile, fasting glucose or HbA1C, blood pressure, and weight	
	Productivity (Work Limitations Questionnaire)	Patient survey
Aim 2	Patient demographics	Patient survey
	Patient comorbidities	Medical record
Aim 3:	CCM costs, patient inpatient, outpatient, ER, Rx use	Medical record/claims data

*Primary outcomes* include changes in mood symptoms and mental health-related quality of life to be ascertained between baseline and 12 months later from the patient surveys. The surveys include the 9-item Patient Health Questionnaire (PHQ-9) (Kroenke et al. 2001; Spitzer et al. 1999) to assess mood symptoms, and health-related quality of life based on the SF-12 Mental and Physical Health Component Scores (MCS/PCS) (Ware et al. 1996).

*Secondary outcomes* will be ascertained from the health plan medical records, claims files, as well as the patient surveys (Table 2). Inpatient hospitalizations (including length of stay) and ER use will be ascertained from Aetna claims data. For hospitalizations, Agency for Healthcare Research and Quality definitions of ambulatory care–sensitive conditions, (http://archive.ahrq.gov/data/safetynet/index.html) will also be used to identify hospitalizations considered preventable.

Measures of guideline-concordant care will be ascertained from Aetna electronic medical and claims records based on diagnostic and treatment codes for medical and psychiatric conditions, as well as utilization, labs, and medication data from a year prior to and up to 24 months after enrollment. Guideline-concordant care measures include previously established metrics for measuring processes of care for mood disorders (Bauer et al. 2009; STABLE: STAndards for BipoLar Excellence. A performance measurement and quality improvement program; 42 CFR Part 425. Medicare Program) as well as indicators from the Accountable Care Organization published rules for performance measures and shared savings (42 CFR Part 425. Medicare Program) (Table 2).

Health care costs will be ascertained from the health plan’s electronic record reviews using a standard assessment tool from the enrollment date to 24 months later (Kilbourne et al. 2010). Cost data will be estimated for each inpatient, ER, and outpatient visit using Current Procedural Terminology (CPT) codes. A relative value unit (RVU) weight will allow for the use of the Medicare Fee Schedule to calculate a standardized cost for each service. Costs incurred in different years will be discounted at an annual rate of 3% and adjusted for inflation and discounted to the baseline (first) year of the study. Preventable hospitalizations over the 12 and 24-month period will be defined using the AHRQ Ambulatory Care Sensitive Condition (ACSC) definition (Prevention Quality Indicators Overview), which identifies conditions for which “good” outpatient care can potentially prevent the need for hospitalization or for which early intervention can prevent complications or more severe disease.

### Analyses

An intent-to-treat analysis will be performed for all analyses. Bivariate baseline analyses will first be conducted to see if randomization was successful by comparing patient demographics and clinical characteristics (e.g., mental health diagnoses) between randomization groups. If there is a lack of equal distribution across groups, these variables will be added as covariates to analyses or propensity scoring will be used. Baseline characteristics will be compared among those enrolled but dropped out over time to those who remained in the study.

Extent and pattern of missing data will be examined for outcome variables as well as for baseline covariates. We expect missing data to be completely at random or at random, in which case the proposed analytical methods will give unbiased estimates of treatment comparison. We will examine how sensitive our conclusions are to potential non-ignorable missingness using a pattern mixture model that will allow us to either model the observed pattern of missingness or change the imputations to represent the likely differences in conditional distributions between observed and missing data (Diggle & Kenward 1994; Little & Rubin 1987). For the latter, we will combine the results from each imputed data using Rubin’s rules (Rubin 1987).

Changes in primary outcomes from baseline to 12 months later (mood symptoms, quality of life) will be treated as continuous variables. Based on pilot data, these outcome measures are expected to be normally distributed. If a continuous outcome exhibits a significant lack of normality, other options will be considered including data transformation, categorization, and non-parametric analyses. We will first visualize the longitudinally assessed outcome data, including plots of cross-sectional means of outcome variables over time. Separate linear mixed-effects models will be run to assess the CCM effects compared to usual care on changes in PHQ-9 and SF-12 MCS scores over 6 and 12-months, adding as covariates the baseline values of the outcome measure, the CCM arm indicator, time, and CCM X time interaction. If CCM by time interaction is not significant, CCM effect averaged over 6 and 12 months will be obtained. Although we expect most study participants will be under the care of one unique provider, the model will account for potential clustering by providers as needed.

For guideline hospitalization and guideline-concordant care, the likelihood of hospitalization (and separately for hospitalizations involving ambulatory care sensitive conditions) and receipt of guideline-concordant treatment from baseline up to 12 months will be determined using a generalized linear mixed-effects model (GLM) with logit link. Similar to our primary hypotheses, linear mixed-effects models will be conducted to determine the effect of CCM versus usual care on changes in the Work and Social Adjustment Scale.

The secondary (exploratory) aim will focus on determining patient factors associated with variation in primary or secondary outcomes. In particular, similar linear mixed-effect and GLM models will be run as described above, but including patient baseline factors such as mood disorder diagnosis, presence of substance use disorders, and provider type (e.g., solo or small group practice) as potential covariates that might explain outcome differences (if any) between those randomized to receive CCM or usual care.

The third exploratory aim will be a cost-effectiveness analysis from the payer perspective involving a comparison of utilization costs of health service providers including the care manager’s time ascertained from time-motion survey. Generalized Linear Models with log link functions will be used to correct for heteroscedasticity and reduce the impact of outliers (Manning & Mullahy 2001). The incremental effectiveness of CCM compared to usual care will be measured by changes in health utilities, assessed using a method described by Zivin (Zivin et al. 2008) and Brazier (Brazier et al. 2005) which translates six of the SF-12 items to changes in health utility (SF-6) based on responses to standard gamble questions given by community members regarding all combinations of possible health states. Cost-effectiveness ratios will be calculated based on the difference in per patient costs and effectiveness of CCM versus usual care. To quantify uncertainty around these ratios, a standard nonparametric bootstrapping approach will be employed. For the business case, additional analyses will be conducted in which changes over time in utilization and costs of inpatient, ER, and outpatient services (medical, psychiatric) will be compared between patients in the CCM or usual care arms over a 2-year period, in order to determine the time dynamics by which the CCM led to changes in health care costs.

### Sample size and power

The study sample size was estimated based on our primary aim and informed by our updated CCM pilot studies that estimate effects on changes in the most conservative outcome change to be expected (Cohen’s D = .36 based on changes in PHQ-9 symptom scores and Cohen’s D = .31 based on changes in SF-12 MCS scores from baseline to 12 months). Assuming a 20% dropout by 12 months, a projected 172 patients enrolled per arm (344 patients total per arm) would provide 82.5% power (two-sided alpha test) to detect the expected between-group difference in mean outcome scores assuming one or two patients per provider with a 0.05 within-provider correlation, and adjustment for multiple comparisons (Bauer et al. 2009).

### Trial status

Staff training and finalization of recruitment procedures occurred in the spring of 2014. Recruitment and CCM implementation will begin by fall of 2014. Recruitment is anticipated to last 18 months, hence allowing for ample time to recruit patients with either bipolar disorder or depression. Years 3-5 will be devoted to follow up data, analyses for secondary aims, as well as study dissemination and implementation activities.

## Discussion

We describe to date one of the first studies to implement a CCM at in a nationwide health plan for patients from small practices, where most mood disorders are seen. CCMs have mostly been implemented at the facility level, and primarily developed for and adopted by larger healthcare organizations. Implementation of evidence-based practices such as the CCM at the health plan level is essential in order to further spread these effective programs to those who need them the most.

Between 50-70% of Americans with mood disorders are managed by commercial insurance plans such as Aetna. A focused implementation of a cross-diagnosis CCM at the national level has implications for the tailoring of evidence-based programs to smaller and rural settings, personalized health care, and implementation of health information technology. As technologies around large databases become more sophisticated and complete, implementation programs that successfully apply these rich resources to helping vulnerable populations will serve as important milestones in the nation’s transition to a more public health model of care.

This study involves a number of strengths, including a groundbreaking health plan- academic partnership, comprehensive data sources, and emphasis on smaller and solo practices. By focusing on measurement dimensions of the Berwick Triple Aim (care, health, and cost), this proposed study is potentially generalizable across complex patient populations (Berwick et al. 2008). Nonetheless, there are key limitations of this proposed design to consider. In addition, while many persons with mood disorders are privately insured under network-model HMOs such as Aetna (Frank et al. 2003), the potential generalizability of this study is restricted to individuals with network-model health plan insurance. This study nonetheless complements a number of initiatives in the public sector that are currently being implemented to increase the uptake of CCMs for mental disorders such as health homes (Collins et al. 2010). Finally, the cost effectiveness analysis is exploratory and not fully powered, but will nonetheless provide valuable information for organizations considering its further adoption.

## Conclusions

Health plan-level CCMs can potentially increase access to evidence –based care and improve outcomes for persons with mood disorders seen by solo or small group practices. This proposed study takes advantage of a unique partnership with a national health plan (Aetna) to develop and implement a CCM designed to improve outcomes for persons with mood disorders for solo or small practices, with an eye towards developing a business case for a generalizable plan-level CCM for chronic disorders. This study will contribute to the evolution of the business case for CCMs in general and enhance the utility of plan-level panel management focused on vulnerable populations across different treatment settings.

## Abbreviations

CCM: Collaborative care model
PHQ-9: Patient Health Questionnaire
SF-12: Short Form Health Survey
ICD-9-CM: International Classification of Diseases, Ninth Revision, Clinical Modification
RA: Research assistant
MCS: Mental Component Score of SF-12
PCS: Physical Component Score of SF-12
CPT: Current procedural terminology
RVU: Relative value unit
ACSC: Ambulatory care sensitive condition
GLM: Generalized linear mixed-effects model
HMO: Health maintenance organization
